# Hip preservation surgery: surgical care for femoroacetabular impingement and the possibility of preventing hip osteoarthritis

**DOI:** 10.1093/jhps/hnu015

**Published:** 2014-10-28

**Authors:** Carl R. Freeman, Michael G. Azzam, Michael Leunig

**Affiliations:** 1. Department of Orthopaedic Surgery, Naval Hospital Jacksonville, 2080 Child Street, Jacksonville, FL 32214, USA; 2. Department of Orthopaedic Surgery, University of Tennessee-Campbell Clinic, 1211 Union Avenue, Suite 510, Memphis, TN 38104, USA; 3. Hip Service, Department of Orthopaedics, Schulthess Clinic, Lengghalde 2, Zurich CH-8008, Switzerland

## Abstract

Despite its widespread usage, the hip preservation surgery can be most accurately described as a hypothesis that surgery can preserve a hip and prevent the need for arthroplasty. This premise has not been fully investigated to date, and there exist few summaries of the underlying evidence in regard to the basis of this terminology. This study seeks to define the hip preservation surgery, and then examines this premise critically in the context of treatment for its most commonly treated condition—femoroacetabular impingement. Finally, we report the current level of preservation of the hip that can be expected with current techniques.

## INTRODUCTION

The field of non-arthroplasty surgical treatment of the adult hip is increasingly being referred to as ‘hip preservation surgery’. This term implies that surgery may increase the longevity of the native, affected hip and prevent or delay the need for arthroplasty. At this time, the term ‘hip preservation surgery’ has not been thoroughly described with respect to osteoarthritis (OA), but there are essentially two means by which it can be defined. The first is a definition using the surgical procedures and techniques (Table I). The second is a definition using the disorders commonly treated (Table II). The most common condition within this field is femoroacetabular impingement (FAI). Thus, the purpose of this review is to examine the underlying premise of ‘hip preservation surgery’ critically in the context of treatment for its most commonly treated condition—FAI, and report the current level of preservation of the hip that can be expected with current techniques. Two hypotheses form the basis of hip preservation surgery in the context of FAI. Those are (i) that FAI causes damage and subsequent OA of the hip and (ii) that surgical treatment can delay or even prevent this process.

**Table I.** Hip preservation surgery

Techniques
PAO
Surgical dislocation of the hip
Proximal femoral osteotomies
Hip arthroscopy
Procedures
Acetabular reorientation
Acetabuloplasty
Labral repair/reconstruction
Cartilage restoration
Femoroplasty
Femoral reorientation

**Table II.** Hip preservation surgery: conditions

Hip dysplasias
FAI
Other hip impingements
Sequelae of Legg-Calvé-Perthes'
Hip cartilage injuries
Coxa valga/vara

### Association between FAI and OA

While it is clear that OA is caused by a cycle of cartilage breakdown, subsequent pro-inflammatory cytokine pathways and additional cartilage changes with a higher susceptibility to breakdown, the initiating event has been poorly understood until recently [1–3]. Ganz *et al.* [4] initially described the modern concept of FAI and theorized that the cartilage damage from FAI initiated the cascade that can lead to early OA. They were the first to describe cam and pincer types of impingement and identified stereotypical patterns of cartilage damage associated with these distinct but not mutually exclusive mechanical disorders. Subsequent research has shown that chondral damage in fact does occur first in the areas of mechanical impingement [5–8], and histological exam has shown that this damage resembles early osteoarthritic changes [9]. While commonly found together, each type of FAI has a distinct pattern of cartilage and labral injury, and irrespective of type, chondral damage begins on the acetabular side of the joint, most commonly in the anterosuperior acetabulum [5–7, 10].

In one of the first studies, Beck *et al.* [5] showed that FAI causes cartilage damage. This study described the patterns of injury associated with isolated cam and pincer lesions. Their study of 302 non-arthritic hips (Tönnis grade <1) treated with surgical dislocation had a total of 26 hips with pure cam lesions and 16 hips with pure pincer lesions, further highlighting the mixed nature of FAI in the majority of cases. The majority of the cartilage damage in the isolated cam group was found in the anterosuperior acetabulum at the 1 o’clock position, and the chondro-labral junction was disrupted in all hips. This cartilage injury was a mix of debonding, cleavage and malacia. Finite element analysis has shown that hips with a center edge (CE) angle >30°, and an alpha angle >50° are exposed to critically higher von Mises stress, which can result in chondral damage and shear forces that can delaminate at the osteochondral junction [11]. Specifically, the greatest osteochondral shear stress and von Mises stress occurs at the anterosuperior acetabulum in hips with cam deformity, supporting cam deformity as the causative factor for cartilage damage in this area [8, 11–13]. Conversely, circumferential labral damage was most common in the pincer impingement group, with the most severe areas between 11 and 1 o’clock, and the acetabular cartilage damage injury was generally restricted to a narrow circumferential band around the acetabular rim [5]. Finite element analysis has shown that the greatest labral stress and deformation occurs in the lower zone of the labrum, adjacent to the chondral labral junction, exposing this area to the greatest risk of soft-tissue injury [12]. Hips with pincer deformity can also demonstrate a ‘contre-coup’ lesion of the posteroinferior acetabulum and femoral head [5].

Magnetic resonance imaging (MRI) and computed tomography (CT) have been used to detect the initial stages of cartilage damage that may lead to eventual OA [14, 15]. Dolan *et al.* [14] reviewed CT scans of 137 patients with MRI-confirmed acetabular labral tears and found that 90% of these patients had structural abnormalities associated with FAI—43% included acetabular retroversion. In a cross-sectional study of 1080 young male asymptomatic Swiss individuals, 244 MRIs were performed. Those with higher alpha angles and cam deformity had significantly higher rates of associated labral damage, impingement pits in the femoral head–neck junction and decreased anterosuperior cartilage thickness [15]. These findings persisted after adjusting for age and BMI, and the majority of the damage on MRI was located in the anterosuperior quadrant. However, 67% of patients without cam lesions also demonstrated labral abnormalities on MRI, highlighting the potential of over-reading MRI alterations and the need for longitudinal studies to determine which labral abnormalities will become symptomatic over time. In regard to rate of progression of OA, Goker *et al.* performed a longitudinal, retrospective radiographic study of 99 patients undergoing total hip arthroplasty (THA) to evaluate quantitative joint space narrowing in the contralateral hip. With a mean follow-up of 104 months, patients either underwent a slow or a rapid rate of decline in joint space width. Progression was linear, and the rate of decline in the first 20 months was predictive of a constant rate. Thus, AP pelvis radiographs may be followed over relatively short periods to identify patients undergoing rapid joint space decline for possible interventions [16].

FAI morphology increases the risk of OA, and this has been validated for cam deformity [8, 17–20]. Less data are available for pincer deformity [5, 21] including acetabular retroversion [22]. In a prospective study of 865 patients, Agricola *et al.* measured alpha angles on weight-bearing AP pelvis radiographs for cam impingement at baseline and 5 years later. Moderate (α angle >60°) and severe (α angle >83°) cam-type deformity correlated with an adjusted odds ratio of 3.67 (95% CI: 1.68–8.01) and 9.66 (95% CI: 4.72–19.78), respectively, for end-stage OA. The combination of severe cam-type deformity and decreased internal rotation at baseline resulted in an even more pronounced adjusted OR of 25.21, and a positive predictive value of 52.6% for end-stage OA. Doherty *et al.* [19] examined radiographs of 1007 patients with hip OA and compared them to 1123 control patients who were undergoing intravenous urography in a case–control study. They found that a pistol-grip deformity of the proximal femur was significantly more common in both the affected and unaffected hips of those patients with OA when compared with the control group. The pistol-grip deformity was also much more common in men (6%) than women (0.4%). Gosvig *et al.* [20] examined 2803 pelvic radiographs from the Copenhagen Osteoarthritis Study, a large, prospective, cross-sectional, population-based study. They found that overall only 6% of men and 2% of women had cam deformities, but 42% of patients who required total hip arthroplasty had evidence of cam deformity. Nicholls *et al.* [18] evaluated AP pelvis radiographs from 1003 women in the Chingford Study Group at 2 and 20 years after enrollment. They found that increased alpha angle and decreased CE angle were independent predictors for future total hip arthroplasty using multivariate analysis. There was a 5.8% increase in THA risk per degree increase in alpha angle, and a 10.5% increase in THA risk per degree reduction in CE angle. Thus, cam deformity is closely associated with increased risk for both OA and the need for THA.

Acetabular retroversion has also been associated with increased risk of OA. Giori and Trousdale [22] examined 131 radiographs taken before THA for primary OA and compared them to 99 pelvis radiographs taken for non-orthopedic reasons. Acetabular retroversion was noted on 20% of hips that went on to have THA, whereas only 5% of the asymptomatic hips had signs of retroversion. In another study utilizing the Copenhagen Osteoarthritis Study cohort, Gosvig *et al.* [21] analyzed pelvic radiographs for abnormalities associated with dysplasia, deep acetabular sockets (such as with coxa profunda or protrusio), pistol-grip deformity and OA. While the presence of acetabular dysplasia was relatively low (4.3% for men and 3.6% of women), there was a relatively high prevalence of deep acetabular sockets in both men and women (15.2% and 19.4%, respectively) and pistol-grip deformity in men (19.6%). Deep acetabular sockets resulted in an adjusted risk ratio of 2.4 for development of concomitant hip OA, and pistol-grip deformity had an adjusted risk ratio of 2.2. This study demonstrates that pincer deformities (deep acetabular sockets) result in an increased risk of subsequent OA. Bardakos and Villar performed a longitudinal, retrospective radiographic study of 43 patients with radiographic signs of OA in the setting of FAI (Tönnis 1 or 2) who had AP pelvis radiographs taken 10 years apart. Twenty-eight hips had progression of arthritis. They concluded that a lower medial proximal femoral angle and a posterior wall sign were predictors of OA development. However, more than a third of their patients had no radiographic progression of arthritis, whereas the rest had a more rapid progression, which could not be explained. This final study may indicate two contrasting natural histories for untreated FAI—one of rapid progression to OA and another with more gradual progression [23].

FAI morphology is prevalent in hips with OA that are undergoing THA, especially those of younger patients [18, 20]. Clohisy *et al.* [24] retrospectively analyzed radiographs of 604 patients under age 50 who were undergoing THA. Of the 337 patients who had OA, 121 of these hips had an etiology other than developmental dysplasia, Legg-Calvé-Perthes' disease or slipped capital femoral epiphysis. These 121 patients were evaluated for structural abnormalities associated with FAI, and 97.5% of these hips had signs of cam, pincer or mixed impingement. When examining the contralateral hips of the 70 patients with adequate serial radiographs, structural abnormalities were seen in all hips, and 37% of these hips underwent subsequent THA at an average of 5.1 years later. Tanzer and Noiseux [7] prospectively examined the history and radiographs of 200 consecutive patients scheduled for THA. Of the 125 patients classified as idiopathic arthritis, all patients were classified as having a pistol-grip deformity of the proximal femur. In addition, when examining the contralateral hip, 17% of patients had a contralateral THA, 55% had a pistol-grip deformity and radiographic evidence of arthritis and 14% had a pistol-grip deformity and no evidence of arthritis. These studies demonstrate a high rate of FAI structural abnormalities in patients undergoing THA, many of whom were previously thought to have ‘idiopathic’ arthritis.

### Surgical treatment of FAI abnormalities

The goal of surgical treatment of FAI, a condition caused by abnormal femoral and acetabular morphology, is to attempt to recreate normal anatomic morphology to interrupt the process of mechanical damage. This is accomplished by rounding the femoral head-neck junction, reducing excessive acetabular rim or reorienting the acetabulum, and/or repairing the acetabular labrum [25–30]. Three major techniques are commonly used to accomplish this goal. Advantages and limitations of each technique are listed in Table III.

**Table III.** Advantages and limitations of each surgical technique

	Advantages	Limitations
Surgical dislocation of the hip	Access to the entire femoral head and neck	Potential complications of symptomatic hardware and non-union
	Optimal visualization for correction of deformity	
	Ability to confirm sphericity with open templates	Increased blood loss
	Treatment of intra-articular cartilage defects	Ligamentum teres disruption
	Open dynamic assessment of impingement	Potential for prolonged rehabilitation
	Ability to perform other correction procedures	
Hip arthroscopy	Minimally invasive	Traction-related complications and nerve injury
	Potential reduced pain	Steep learning curve
	Can be an outpatient procedure	Incomplete access and correction of deformity
	Potentially faster rehabilitation	Inability to directly confirm restoration of sphericity/offset
	Potentially reduced soft-tissue injury	Potential for iatrogenic chondral injury
		Fluid extravasation and abdominal compartment syndrome
		Portal complications (lateral femoral cutaneous nerve injury)
PAO	Ability to change acetabular orientation	Very invasive, with a relatively high rate of complications
	Can treat pincer FAI without reducing coverage	Increased blood loss
	Can address dysplasia or severe acetabular retroversion	Much slower rehabilitation
		Long learning curve
	Ability to perform other correction procedures	
		Table modified from Zaltz *et al.* [32]

The first technique is surgical dislocation of the hip. This technique allows for safe open exposure of the entire femoral head and circumferential access to the acetabulum [31]. Surgical dislocation is ideal for cases of FAI that involve large cam bumps, especially when large, lateral cam bumps are suspected to be the major morphological culprit. Another indication for surgical dislocation in the treatment of FAI is in the case of global pincer impingement, such as the case of coxa profunda, protrusio or a circumferential ossified labrum. The main advantages of surgical dislocation over other techniques are (i) the exposure of the entire femoral head and acetabulum, which gives unobstructed access to reshape both the acetabular rim and the femoral head, (ii) ability to confirm sphericity of the femoral head-neck junction and (iii) ability to perform other procedures, such as cartilage restoration procedures [32].

In the second technique, arthroscopic hip surgery, arthroscopic instruments are used to reshape the acetabular rim and femoral head-neck junction and repair the labrum through several small incisions. This technique has dramatically increased in popularity over the past decade. Its main advantage is the reduced level of invasiveness compared with surgical dislocation and thus quicker rehabilitation. However, the major disadvantage is the limited region of access with arthroscopy. Specifically, surgical access during hip arthroscopy is limited to the anterior and anterolateral regions of the extra-articular hip (referred to as the peripheral compartment). It is worth reiterating that this is the region where the vast majority of FAI pathology is found. It is also worth noting that some surgeons perform femoroplasty through a small ‘mini-open’ direct anterior or anterolateral incision in combination with hip arthroscopy.

The third technique, reverse periacetabular osteotomy (PAO), can be used in cases of severe retroversion of the hip [33, 34]. This procedure allows the surgeon to reorient the acetabulum around the femoral head, so that over-coverage on the anterior side can be rotated such that the anterior coverage is reduced while the posterior coverage is increased, removing an area of pincer conflict. In addition, an anterior capsulotomy can be performed, and a cam lesion—if present—can be reshaped as well. This procedure is exceptionally versatile because it allows for the removal of a pincer lesion without reducing acetabular coverage, and additionally provides an opportunity to address femoral-sided deformity. However, the disadvantage of reverse PAO is that it is an invasive procedure with a comparatively longer recovery, and the potential for overcorrection leading to posterior impingement.

To determine the extent to which surgery interrupts FAI, thereby preserving the hip, a review of the outcomes literature was performed.

## METHODS

A PubMed search was conducted from inception to 24 February 2014, to find studies on the treatment of FAI using terms relevant to this disorder. This search resulted in 923 articles. Studies were limited to clinical trials, prospective series and retrospective series. Articles were excluded if articles (i) did not include surgical treatment, (ii) were exclusively focused on pediatric patients, (iii) did not have outcomes data or (iv) had a mean follow-up <2 years. The search results were reviewed, and only 63 involved surgical treatment, all others were excluded. Of these, only 41 had outcomes data, and only 22 had mean follow-up >2 years. The abstracts of the remaining articles after exclusion were reviewed for relevant studies not covered by the included articles. We also searched the Cochrane reviews database to find meta-analyses published in the last 15 years, which resulted in no articles. Two articles were excluded for poor methodology, and one was excluded due to the use of a non-standard outcomes measure. The remaining 22 articles are the substance of this study. There was no external funding support for this study.

## RESULTS

Due to the fact that FAI is a diagnosis just over a decade old with treatments that are continuing to evolve, well-designed longitudinal long-term outcome studies on outcomes of treatment are not yet available. Thus, the only evidence available to date is mid-term and short-term data, with low level of evidence as most were case series. Furthermore, the majority of the evidence that met the inclusion criteria is Level IV (21 studies), with only three Level III studies and one Level II study (Table IV). Despite the weakness in level of evidence and long-term data, examination of the available evidence provides information about the trends and successes of surgical treatment for FAI. We found two data points to be especially relevant and broadly reported when assessing studies involving surgical care for FAI. These are (i) improvement in hip pain and (ii) function and progression to arthroplasty.

**Table IV.** Outcome studies

	Study [reference]	Hips	Level of evidence	Study years	Mean follow-up	Approach	Mean age	Outcome score	Pre-operative score	Post-operative score	THA	THA%
Short-term outcomes	Espinosa *et al*. 2006 Group 1 [27]	25	3	1999–2001	24 months	Dislocation with labral resection	30	Merle d’Aubigné	12	15	0	0.0%
	Espinosa *et al.* 2006 Group 2 [27]	35	3	2001–02	24 months	Dislocation with labral repair	30	Merle d’Aubigné	12	17	0	0.0%
	Graves and Mast 2009 [37]	48	4	2000–03	38 months	Dislocation	33	Merle d’Aubigné	13	17	0	0.0%
	Philippon *et al.* 2009 [39]	112	2	2005	28 months	Arthroscopy	40	mHHS	58	84	10	8.9%
	Brunner *et al.* 2009 [40]	53	4	N/A	28 months	Arthroscopy	42	NAHS	54	86	N/A	
	Horisberger *et al.* 2010 [41]	105	4	2004–07	29 months	Arthroscopy	46	NAHS	57	85	9	8.6%
	Chiron *et al.* 2012 [47]	118	3	2005–10	26 months	Open with labral excision	34	mHHS, NAHS, WOMAC	63,59,33	93,91,5	4	3.4%
	Parvizi *et al.* 2012 [48]	156	4	2006–11	27 months	Mini-open with labral repair	32	mHHS, UCLA,WOMAC	58,6,45	86,8,11	11	7.1%
	Larson *et al.* 2012 Group 1 [46]	44	3	2006–07	42 months	Arthroscopy—labral debridement	32	mHHS	65	85	0	0.0%
	Larson *et al.* 2012 Group 2 [46]	50	3	2006–07	42 months	Arthroscopy—labral repair	28	mHHS	65	94	1	2.0%
	Palmer *et al.* 2012 [49]	210	4	2005–08	46 months	Arthroscopy	40	NAHS	56	78	0	0.0%
	Clohisy *et al.* 2010 [43]	35	4	2003–05	26 months	Arthroscopy with limited open	34	mHHS	64	87	0	0.0%
	Byrd and Jones 2009 [29]	100	4	2003–06^a^	24 months	Arthroscopy	34	mHHS	65	87	0	0.0%
	Nho *et al.* 2011 [44]	47	4	2007–08	27 months	Arthroscopy	23	mHHS	69	86	0	0.0%
	Philippon *et al.* 2012 [45]	153	4	2006–08	36 months	Arthroscopy	57	mHHS	58^b^	84^b^	31	20.3%
	Weighted mean of short-term outcomes	1291			29 months		36				66	5.1%
Mid-term outcomes	McCarthy *et al.* 2011 [50]	111	4	1989–97	13 years	Arthroscopy	39	NAHS	NA	87^b^	49	44.1%
	Byrd and Jones 2009 [35]	31	4	1993–98	10 years	Arthroscopy	46	mHHS	56	81	7	22.6%
	Meftah *et al.* 2011 [51]	50	2	1996–2003	8.4 years	Arthroscopy	40	mHHS	79	92	2	4.0%
	Murphy *et al.* 2004 [52]	23	4	1990–2002	5.2 years	Open	35	Merle d’Aubigné	13	17^a^	7	30.4%
	Naal *et al.* 2012 [53]	233	4	2003–06	5.1 years	Open	30	WOMAC	NA	10	7	3.0%
	Steppacher *et al.* 2014 [54]	97	4	2001–13	5 years	Dislocation with labral repair	32	Merle d’Aubigné	15	17	7	7.2%
	Laude *et al.* 2009 [55]	94	2	1999–2004	4.8 years	Combined	33	NAHS	55	84	11	11.7%
	Beck *et al.* 2004 [56]	19	4	1996–97	4.7 years	Open	36	Merle d’Aubigné	14	17	5	26.3%
	Weighted mean of mid-term outcomes	658			6.8 years		34				95	14.4%

N/A, not available.

^a^Estimated, ^b^excluding patients who went on to receive THA.

### Short-term outcomes

Seventeen short-term studies were included in this review [27, 29, 35–45]. Combined, these reflect the results of 1251 hips with a mean follow-up of 29 months, and patients with a mean age of 35 years. The most commonly measured outcome score was the modified Harris hip score (mHHS), which had a mean improvement of 24 (37%) in these studies. Several studies also included the Merle d’Aubigné score, which had a mean improvement of four (33%), and the non-arthritic hip score (NAHS), which had a mean improvement of 30 (54%). This reflects a sizable improvement in hip pain and function. These studies found that the vast majority of surgically treated patients experience improvement in symptoms at 2–3 years follow-up as measured by outcomes questionnaires. Seventy patients (6%) required conversion to THA. Thus, very few patients went on to receive arthroplasty in the short term.

Two short-term outcome studies were especially noteworthy. The first was performed by Philippon *et al.* [45], and examined the results of an older age group, mean age 57 years (range 50–77) treated with arthroscopic debridement and osteochondroplasty. In total, 31 hips (20%) went on to receive arthroplasty at a mean of 1.6 years. Outcome scores of the surviving hips—excluding those that went on to receive arthroplasty—showed improvement from 58 on the mHHS pre-operatively to 84 at mean follow-up of 36 months. This study shows that there may be utility in considering surgery to address FAI even for an older patient group; one in which arthroplasty would have traditionally been the only surgical treatment available.

In the second study, Larson *et al*. [46] examined outcomes of 94 patients with FAI who underwent either labral debridement or labral refixation in a retrospective cohort study. The cohort who underwent labral debridement included patients who were treated before labral refixation was routine, but had labral tears that would have been amenable to refixation. At a mean 3.5-year follow-up (range 24–72 months), HHS, SF-12 and VAS pain scores were significantly improved in the refixation group compared with the debridement group, with no difference in reoperation rate. At most recent follow-up, good to excellent results (HHS >80) were noted in 68.2% of hips in the debridement group, and 92% of hips in the refixation group. This study suggests that labral refixation results in superior functional outcome scores compared with debridement.

### Mid-term outcomes

Several retrospective, mid-term studies were included in this review (Table IV) [27, 29, 35, 37, 39–41, 43–56]. These studies reflect the results of 658 hips with a mean follow-up of 6.8 years, and include patients with a mean age of 34 years. Outcome measures showed high rates of function and satisfaction, with low rates of pain. All studies found a mean increase in outcomes scores. Ninety-five hips (14%) required conversion to THA.

Three studies involving arthroscopic treatment are available. McCarthy *et al.* [50] performed arthroscopic debridement on 111 hips, mean age 39 years (range 26–52), with minimum follow-up of 10 years. About 44% went on to have arthroplasty, at an average of 4.8 years. Healthier cartilage scores were associated with survival of native joints, while higher age and worse cartilage scores predicted eventual arthroplasty. Byrd and Jones [35] treated 31 hips with arthroscopic labral debridement. The study included only hips with labral tears. The mean age was 46 years old (range 17–84), and follow-up was 10 years. The study, which used a prospective mHHS to measure outcomes, found that a median pre-operative score of 52 increased to 81 at 10-year follow-up. Arthritic changes were associated with poor outcomes. Meftah *et al.* [51] treated 50 hips with arthroscopic labral debridement, and included only patients with labral tears. Mean age was 40 years. Pre-operative HHS was 79 which improved to 92 at a mean follow-up of 8.4 years (range 7–13.6) post-operatively. Two cases went on to receive arthroplasty 4.5 and 5.2 years post-operatively. Only 19% of patients with arthritis experienced good or excellent outcomes.

Five mid-term studies of open surgical results are available. Murphy *et al.* [52] treated 23 hips with FAI via surgical dislocation and osteochondroplasty. Mean age was 35 years (range 17–54), and mean follow-up was 5.2 years (range 2–12). Outcomes were measured with Merle d’Aubigné score which improved from 13.2 to 16.9 among surviving hips. Pre-existing OA and acetabular dysplasia were associated with progression to arthroplasty in seven hips. Naal *et al.* [53] treated 233 hips with FAI via surgical dislocation, osteochondroplasty and labral repair or debridement. Mean age was 30 years (range 14–55), and mean follow-up was 5.1 years (range 2–10). Outcomes were measured with the Western Ontario and McMaster Universities Osteoarthritis Index (WOMAC) and the Hip Outcome Score, but pre-operative scores were not obtained. About 82% of patients were satisfied or very satisfied, and outcome scores revealed high rates of function and low pain levels at follow-up. They reported a very low rate of conversion to arthroplasty, 3%. Steppacher *et al.* [54] examined the results of surgical hip dislocation with femoral and acetabular osteoplasty with labral reattachment for FAI in a retrospective cohort study of 75 hips (97 patients) at 6 years mean follow-up (5–7 years). At final follow-up, there were significant improvements in hip internal rotation, abduction and Merle d’Aubigné-Postel scores. Kaplan–Meier analysis showed 91% survivorship without progression of OA or conversion to THA, and good to excellent clinical scores at final follow-up. Excessive anterior rim trimming, pre-operative OA, increased age and higher weight were associated with early failure using multivariate analysis. This study shows excellent survivorship after surgical hip dislocation and improved functional outcomes at mid-term follow-up.

Laude *et al.* [55] treated 94 hips with arthroscopy followed by open anterior approach with osteochondroplasty. Mean age was 33 years (range 16–56), and mean follow-up was 4.8 years (range 2.4–8.7). NAHS was used to measure outcome, which improved from a mean pre-operative score of 55, to 84 in follow-up. Eleven hips went on to receive arthroplasty, all of which had ‘advanced chondral damage’. Beck *et al.* [56] treated 19 hips with surgical dislocation and osteochondroplasty. Mean age was 36 years (range 21–52), and mean follow-up was 4.7 years (range 4.2–5.2). Merle d’Aubigné score was used to measure outcomes, which improved from mean 14.1 pre-operatively to 16.5 at final follow-up. Five hips went on to receive arthroplasty at an average of 3.1 years. Severe labral and chondral degeneration were associated with progression to arthroplasty.

## DISCUSSION

There is strong evidence that surgical treatment results in a reduction or prevention of FAI and theoretically in preservation of the hip. This is evidenced by the improvement in outcome scores and the low rate of progression/conversion to THA shown by the included studies. Mean hip outcome scores improved in every study suggesting that surgery for FAI can be reasonably expected to result in improvement in pain and function and probably in disruption of the cycle of chondral damage caused by FAI. Next, the mean rate of conversion to THA was 6% in the short-term and 14% in the mid-term. This indicates that hips treated with surgery for FAI have a low rate of failure. However, a wide variation exists regarding the rates of conversion to arthroplasty, 0–44%. This variation may be due to the improved success of evolving techniques, but it may also reflect differing patient populations. Furthermore, this is a source for optimism that sustained investigation will identify increasingly successful techniques and surgical indications and may identify patients at risk of high failure rates.

Several prognostic patterns emerge in this review and are supported in the literature. Good outcomes are associated with (i) younger age (i.e. <40 years), (ii) limited or no pre-operative cartilage damage or arthritic changes and (iii) labral refixation in the setting of labral pathology [43]. Meanwhile poor outcomes and conversion to arthroplasty are associated with (i) older age, (ii) pre-operative cartilage damage and arthritic changes and (iii) increased pre-operative pain [43]. Almost all studies show high rates of improvement in outcomes scores with surgical treatment, however, these scores may not adequately reflect patient perceptions. Impellizzeri *et al*. showed that while 85% of patients experience improvement in hip outcome scores, only ∼70% exceed a threshold that would be considered a good or excellent result [57], and this is the success rate that should be considered when counseling a patient regarding the decision for surgical treatment. Furthermore, they found that younger patients have a higher threshold of satisfaction in terms of pain and function than do patients with other diseases.

Three recent systemic reviews have come to a similar conclusion in regard to the efficacy of surgery for FAI [43, 58, 59]. Clohisy *et al.* [43], Bedi *et al.* [58] and Stevens *et al.* [59] each completed a comprehensive, systematic, literature review and concluded that surgical treatment for FAI resulted in high rates of success in terms of reduced pain and increased function as measured by validated outcomes scores. However, all noted the significant lack of high level evidence, with each study predominantly composed of retrospective series. The study by Stevens *et al.* [59] graded the level of evidence in relationship to several indications for hip surgery. Their systematic review noted the high rate of good or excellent results from surgical treatment of FAI compared with a much lower rate of good or excellent results in surgical cases where FAI was not directly addressed surgically. They also noted the low levels of evidence available, and concluded that there exists ‘fair evidence’ in support of recommending surgical intervention to address FAI. All three studies observed the inverse relationship between cartilage damage and good outcomes, and the negative prognostic value of osteoarthritic changes. The major weaknesses of these studies and this study are the limited data available and the paucity of level 1 or 2 evidence.

Several surgical trends were notable in our literature review. First, surgeons who treat FAI are increasingly choosing less invasive techniques such as hip arthroscopy, and thus pushing further the limits imposed by arthroscopic surgery. Second, there currently exists a wide variability in the treatment of FAI, with individual surgeons treating the condition uniquely. A major focus on research in this field is to determine which techniques such as labral repair and microfracture are most effective, leading to a more consistent and successful surgical approach to FAI. Current evidence has established relatively few contraindications, and researchers are seeking to determine which patients are at risk for a poor result, and therefore more specifically define contraindications for surgical treatment of FAI. Finally, the surgical techniques being used today are distinct from those being used only 5–10 years ago introducing further uncertainty. Thus, with regard to the most current techniques, the best measures of the potential of joint preservation may lie in the results of short-term studies, especially in terms of pain relief, which seems to inversely correlate with continued joint damage.

To definitively state that surgical treatment for FAI prolongs the lifetime of a native hip, one would need longitudinal long-term survival data, which does not exist yet, but there certainly exists evidence that hips treated surgically for FAI have a low rate of failure and conversion to arthroplasty. Furthermore, there is good evidence supporting the effectiveness of surgical treatment in reducing hip pain associated with FAI, allowing patients to sustain higher activity levels. Both mid-term and short-term studies have shown that surgical treatment is associated with a low rate of progression of radiographic signs of OA [43]. Failures tend to be the result of inadequate correction of deformity, unaddressed labral pathology, adhesions, pre-existing cartilage damage and progression of OA. The results of the available evidence gives reason for considerable optimism that surgery for FAI will result in preservation of the hip.
